# Identifying breast cancer subtypes associated modules and biomarkers by integrated bioinformatics analysis

**DOI:** 10.1042/BSR20203200

**Published:** 2021-01-08

**Authors:** Yanwei Wang, Yu Li, Baohong Liu, Ailin Song

**Affiliations:** 1 Department of General Surgery, Lanzhou University Second Hospital, Lanzhou 730030, Gansu, People’s Republic of China; 2 State Key Laboratory of Veterinary Etiological Biology, Key Laboratory of Veterinary Parasitology of Gansu Province, Lanzhou Veterinary Research Institute, Chinese Academy of Agricultural Sciences, Lanzhou, Gansu Province, People’s Republic of China

**Keywords:** Biomarkers, breast cancers, coexpression, modules

## Abstract

Breast cancer is the most common form of cancer afflicting women worldwide. Patients with breast cancer of different molecular classifications need varied treatments. Since it is known that the development of breast cancer involves multiple genes and functions, identification of functional gene modules (clusters of the functionally related genes) is indispensable as opposed to isolated genes, in order to investigate their relationship derived from the gene co-expression analysis. In total, 6315 differentially expressed genes (DEGs) were recognized and subjected to the co-expression analysis. Seven modules were screened out. The blue and turquoise modules have been selected from the module trait association analysis since the genes in these two modules are significantly correlated with the breast cancer subtypes. Gene Ontology (GO) and Kyoto Encyclopedia of Genes and Genomes (KEGG) pathway enrichment show that the blue module genes engaged in cell cycle, DNA replication, p53 signaling pathway, and pathway in cancer. According to the connectivity analysis and survival analysis, 8 out of 96 hub genes were filtered and have shown the highest expression in basal-like breast cancer. Furthermore, the hub genes were validated by the external datasets and quantitative real-time PCR (qRT-PCR). In summary, hub genes of Cyclin E1 (CCNE1), Centromere Protein N (CENPN), Checkpoint kinase 1 (CHEK1), Polo-like kinase 1 (PLK1), DNA replication and sister chromatid cohesion 1 (DSCC1), Family with sequence similarity 64, member A (FAM64A), Ubiquitin Conjugating Enzyme E2 C (UBE2C) and Ubiquitin Conjugating Enzyme E2 T (UBE2T) may serve as the prognostic markers for different subtypes of breast cancer.

## Introduction

Breast cancer, which affects approximately one of every nine women globally, is one of the most widespread cancers among female malignant tumors [1]. It is well known that breast cancer has high heterogeneity at the molecular level. According to the PAM50 molecular typing model, there are luminal A subtype, luminal B subtype, human epidermal growth factor receptor 2 (her2) positive subtype, and basal-like breast cancer subtypes [2,3]. Breast cancer is a complex disease with changed genetic and molecular characteristics. Understanding the nature of these changes can provide opportunities for an individualized approach to treatment. At the present time, whole genomic analysis is one of the most efficient ways of studying the diseases. Many researchers focus on prognostic or therapeutic markers for identification using differential expression analysis. In addition to differential gene expression, gene co-expression networks have been extensively explored for high-throughput sequencing data analysis [4]. One of the most frequently applied gene co-expression analysis methods is Weighted gene co-expression network analysis (WGCNA) which can explore the patterns of co-expressed genes from a systematic level. This method provides unique insight into the structure and behavior of molecular interactions than individual gene. WGCNA has been used in many studies to identify disease-related modules and marker genes including cancer [5–8]. In the present study, RNA sequencing profiles of breast cancer from luminal A subtype, luminal B subtype, her2 positive subtype, and basal-like subtype were downloaded from The Cancer Genome Atlas (TCGA) database [9]. Further, differentially expressed genes (DEGs) were screened for the pairwise comparisons of the four groups (six comparison pairs). After screening, the union of the six lists of DEGs was subjected to the WGCNA to identify gene co-expression modules. The module trait association analysis and the gene functional enrichment reveals that the blue module plays a pivotal role across various subtypes of breast cancer. Further, hub genes for the blue module were identified and validated by the external database, constituting potential prognostic and therapeutic biomarkers for breast cancer subtypes.

## Materials and methods

### Data process

The breast cancer RNA-Seq data and the corresponding clinical data were downloaded from the TCGA database [9].

### Screening of DEGs

The edgeR package [10] was performed for differential expression analysis for breast cancer subtype pairs. Genes with fold changes ≥ 2, and fdr values <0.01 that adjusted for multiple testing from *P*-values by the Benjamini–Hochberg adjustment method [11] were identified to be differentially expressed.

### WGCNA

A scale free, co-expression network was built by the R package WGCNA on the ground of RNA-Seq data. In this network, the interactions between gene pairs were represented by the Pearson’s correlation coefficient values. Subsequently, the matrix of network topological overlap measure (TOM) was calculated to measure the connectivity of the pair of genes [12]. Hierarchical clustering of average linkage was preformed to identify gene co-expression modules on the basis of the network topology overlap [13]. The subtype representative modules were screened out on the ground of relationships between the modules and external clinical traits (correlation between the eigengene and sample subtypes) and the correlations between gene significance (GS) and module membership (MM) values. Significantly high correlation with *P*-value <0.01 for GS and MM values together with the correlation >0.75 between the module eigengene (ME) and the external trait were used to identify the subtype representative modules. Hub genes were recognized by GS > 0.2 and MM > 0.8 in a specific module.

### Functional enrichment analysis for DEGs

The R package clusterProfiler process [14] was performed for the gene functional enrichment including gene ontology (GO) and KEGG pathway using fdr < 0.05 as the cutoff criteria.

### Survival analysis

The website of GEPIA (http://gepia.cancer-pku.cn/) [15] was applied to analyze the RNA transcriptional data of tumors against normal samples in TCGA database (https://portal.gdc.cancer.gov/). The survival analysis of the hub genes was performed in GEPIA in which the samples were divided into two groups according to the gene expression level. Then the log-rank test algorithm was performed to determine the significance of expression for specific gene on survival. The log-rank test (*P*-value <0.05) was used to determine whether the expression of the hub gene was correlated with the patient’s survival rate.

### Validation of hub genes by independent dataset and quantitative real-time PCR

The differential expression of hub genes were validated in an independent dataset with accession number of GSE45827. There are 130 primary invasive breast cancer (41 Basal like, 30 Her2, 29 Luminal A and 30 Luminal B subtype) as well as 11 normal tissue samples. The survival analysis for hub genes were validated by Kaplan–Meier Plotter (https://kmplot.com/analysis/) which is an online tool to discover and validate the survival biomarkers [16]. In this study, we validated the vital genes by gene chip datasets. Furthermore, we used quantitative real-time PCR (qRT-PCR) to explore the expression level of hub genes. Human breast cancer MCF-7 cells were grown in Dulbecco’s modified Eagle’s medium (DMEM) added with 10% fetal bovine serum. Human normal MCF-10A cells were maintained in the mammary epithelial growth medium DMEM-F12 containing insulin, hydrocortisone, epidermal growth factor, horse serum, and cholera toxin. All cells were incubated at 37°C in a humidified chamber with 5% CO_2_. Total RNA was extracted from cells using RNAiso Plus (Takara, Japan). Complementary DNA (cDNA) was synthesized using the One-Step PrimeScript miRNA cDNA Synthesis Kit (Takara, Japan) according to the manufacturer’s instructions. qRT-PCR was performed using SYBR Premix ExTaq II (Takara, Japan). The relative expression of genes were calculated using the 2^−ΔΔ*C*_t_^ method, normalized to GAPDH, respectively. The primers used in the present study are shown in Supplementary Table S1.

### Functions exploration for each hub gene in breast cancer

The functions for the hub genes were evaluated by the database of GeneMANIA (http://www.genemania.org) which features several bioinformatics methods: physical interaction, gene coexpression, gene colocation, gene enrichment, and website prediction [17,18]. In order to explore the functions for each hub gene in breast cancer, samples of dataset GSE45827 were divided into low (*n*=65) and high (*n*=65), according to each hub gene expression. The R package limma was used to identify DEGs between the two groups. DEGs with absolute log2FC > 1 and FDR < 0.01 were considered significant. GO enrichment, KEGG pathway analysis and single gene gene-set enrichment analysis were performed using R package clusterProfiler [14]. FDR < 0.05 was considered as significantly significant.

## Results

### DEGs screening

Using the edgeR package, with threshold values of adjusted *P*-value (FDR) <0.01 and fold change ≥ 2, we identified the DEGs between each breast cancer subtype groups. A total of 6315 DEGs were identified by the six pairwise comparisons of the samples from the luminal A subtype, the luminal B subtype, the her2 positive subtype, and the basal-like subtype of breast cancer (Table 1; Supplementary Figure S1).

**Table 1:** Comparison of DEGs between various groups

DEG set	DEGs
**LumA_vs_LumB**	939
**LumA_vs_Her2**	2062
**LumA_vs_Basal**	3992
**LumB_vs_Her2**	1573
**LumB_vs_Basal**	3598
**Her2_vs_Basal**	2467
**Total**	6315

### Construction of the co-expression network and identification of modules

**Figure 1:**
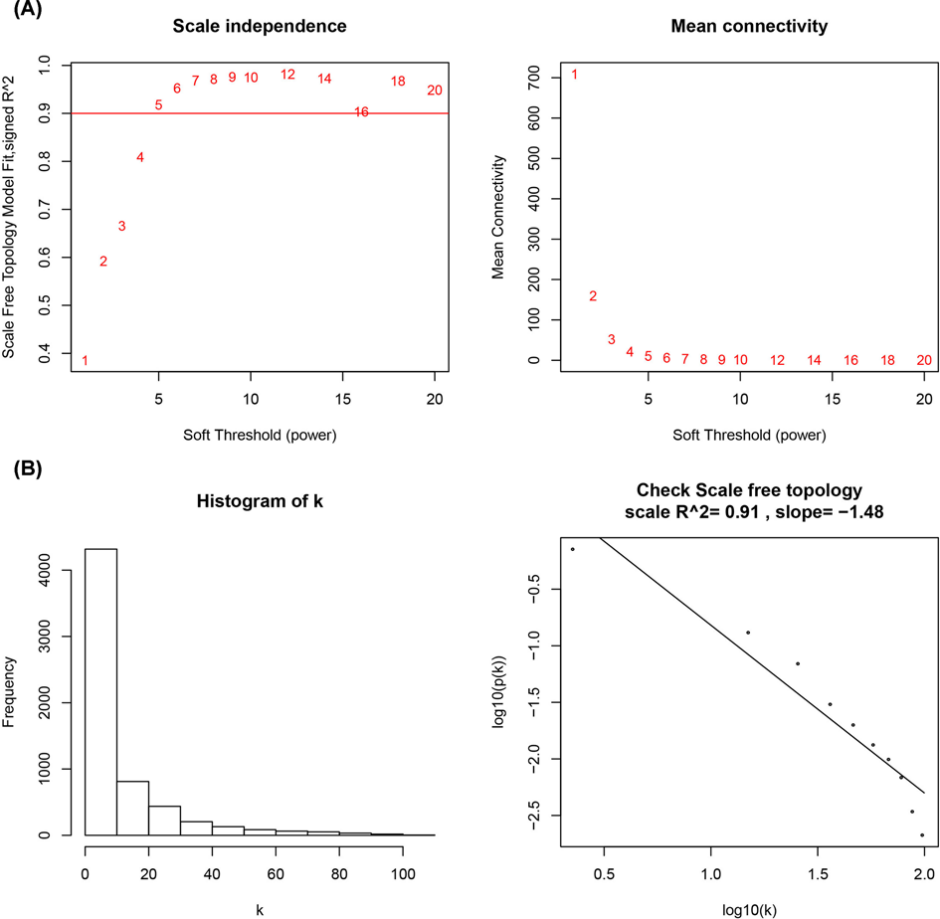
The parameter selection for the weighted gene co-expression network (**A**) The scale-free fitting index of the soft threshold power indicates that 5 is the most suitable power value. (**B**) The node degree distribution.

**Figure 2:**
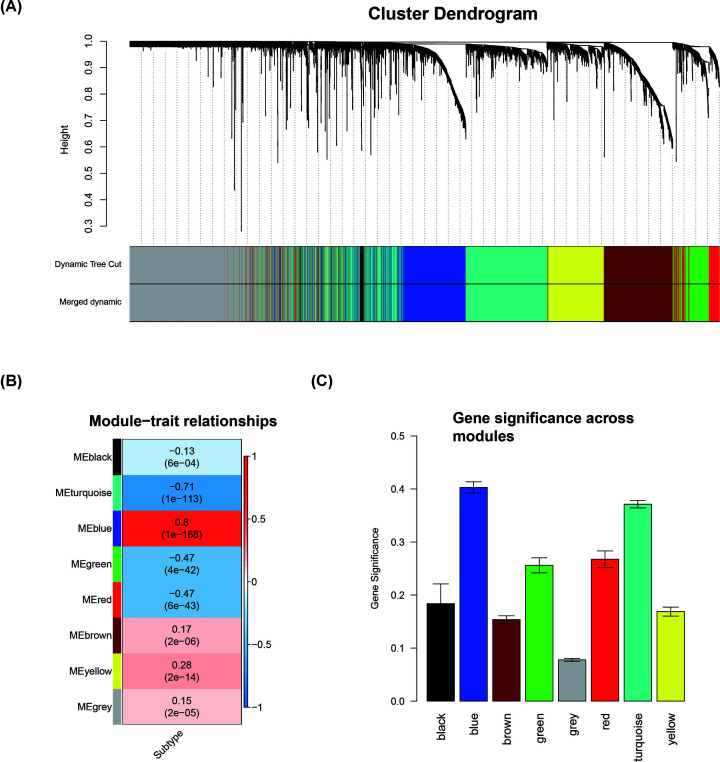
Identification of modules associated with breast cancer subtype (**A**) Cluster dendrogram of all DEGs clustered on a dissimilarity measure. (**B**) Heatmap of the correlations between MEs and the trait for breast cancer subtype. (**C**) Error bars of GS for various models.

**Figure 3:**
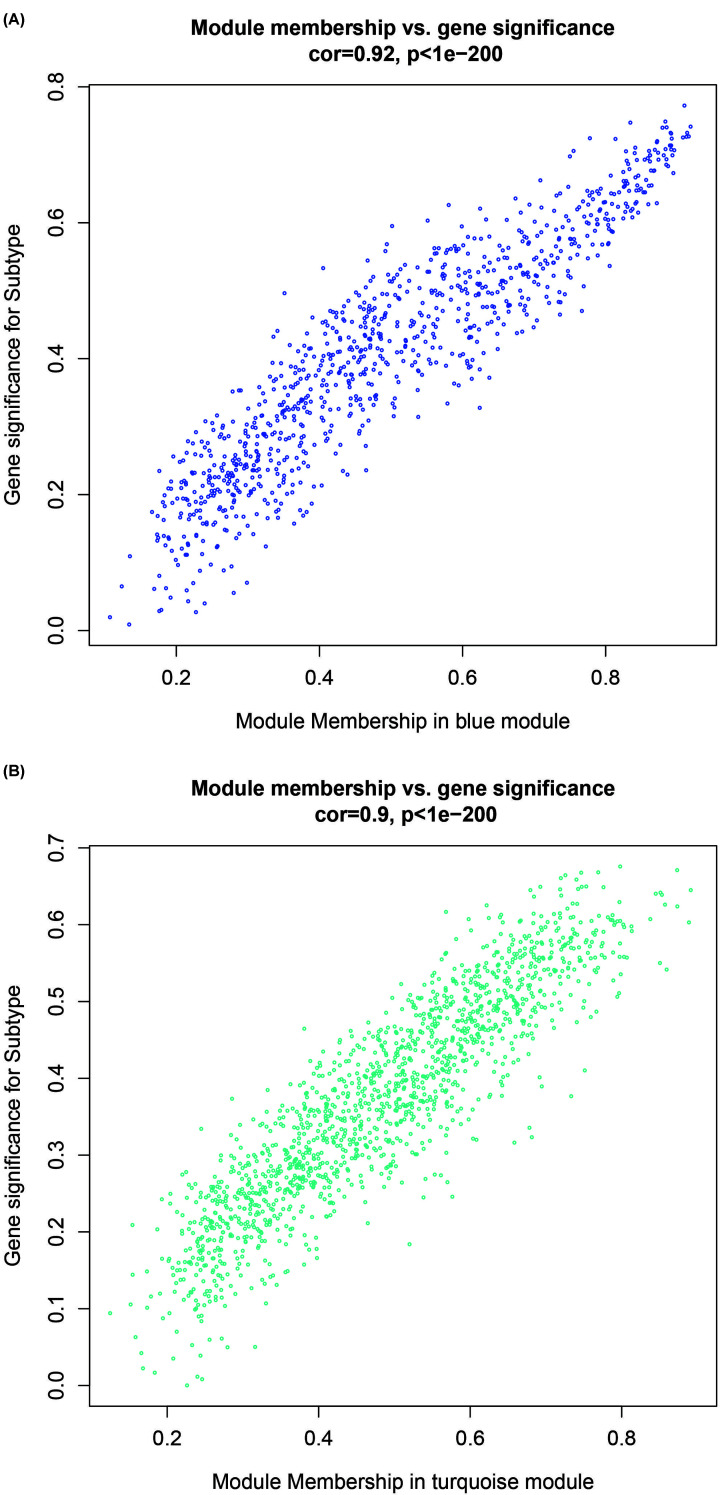
The scatter plot for modules and eigengenes (**A**) The scatter plot of the turquoise MEs. (**B**) The scatter plot of the blue MEs.

WGCNA was performed to recognize modules connected with a variety of breast cancer subtypes. The 6315 DEGs were subject to the co-expression analysis. We analyzed the soft threshold power of the network topology with a β value from 1 to 20, and calculated the scale independence and mean connectivity of the co-expression network. Based on the research data, a suitable threshold of 5 was selected (Figure 1A). The node degree distribution was calculated according to a power-law distribution, indicating that the network was scale-free (Figure 1B). Next, the β = 5 was used to produce a hierarchical clustering gene tree (Figure 2A). The genes’ expression level for a module is represented by ME which is the principal component for a specific module. There are two methods testing the interaction between each module and the corresponding breast cancer subtypes. And two modules were screened out with significant correlations with different cancer subtypes (*P*<0.01 and abs |correlation| > 0.75). Blue (R = 0.8; *P*<1e-168; Figures 2B,C and 3A) and turquoise (R = −0.71; *P*=1e-113; Figures 2B,C and 3B) modules were selected as the key modules associated with the breast cancer subtypes by module–trait association analysis using the Spearman correlation method. Figure 3A,B demonstrate the interactions between the modules turquoise and blues as well as the genetic significance.

### Functional enrichment analysis of genes within the identified modules

**Figure 4:**
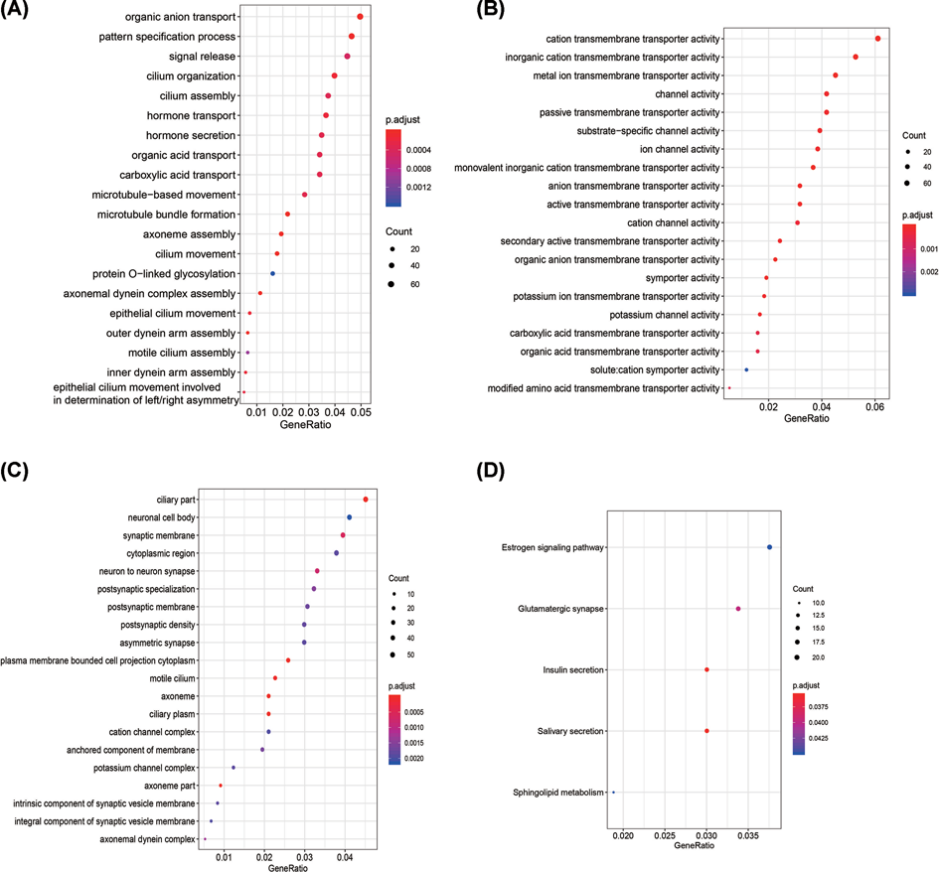
GO enrichment analysis and KEGG pathway analysis of genes in the turquoise module (**A**) The diagram of genes for GO enrichment analysis at the BP level. (**B**) The diagram of genes for GO enrichment analysis at the MF level. (**C**) The diagram of genes for GO enrichment analysis at the CC level. (**D**) The diagram of genes for KEGG pathway enrichment analysis.

**Figure 5:**
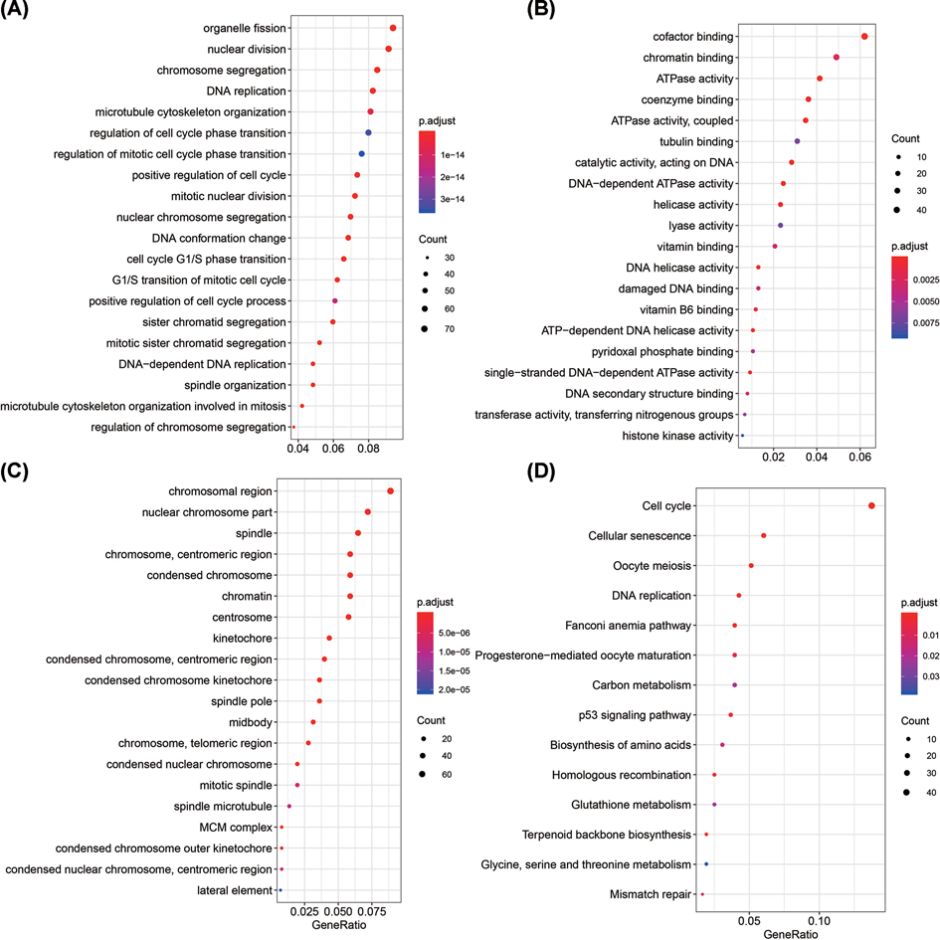
GO enrichment analysis and KEGG pathway analysis of genes in the blue module (**A**) The diagram of genes for GO enrichment analysis at the BP level. (**B**) The diagram of genes for GO enrichment analysis at the MF level. (**C**) The diagram of genes for GO enrichment analysis at the CClevel. (**D**) The diagram of genes for KEGG pathway enrichment analysis.

Gene functional enrichment was applied to the turquoise and blue modules. We observed that genes in the turquoise group are involved in hormone transport, hormone secretion, the estrogen signaling pathway, and cation transmembrane transporter activity related functions (Figure 4). The functional enrichment results demonstrate that the genes in the blue module are significantly associated with cancer such as cell cycle, oocyte meiosis, DNA replication, p53 signaling pathway, and so on (Figure 5).

### Survival and expression analysis of the hub genes

**Figure 6:**
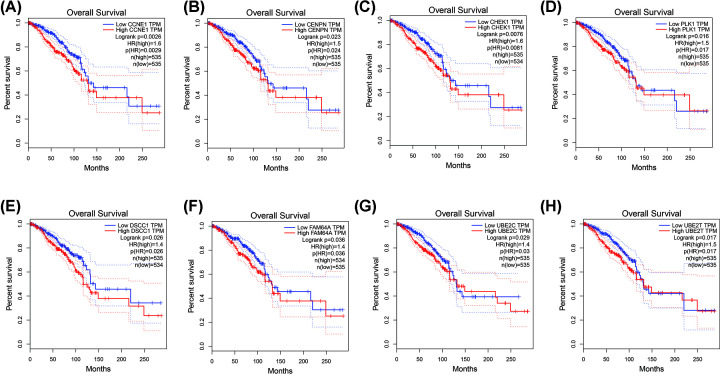
Survival analysis of the hub genes (**A**) Survival analysis for CCNE1. (**B**) Survival analysis for CENPN. (**C**) Survival analysis for CHEK1. (**D**) Survival analysis for PLK1. (**E**) Survival analysis for DSCC1. (**F**) Survival analysis for FAM64A. (**G**) Survival analysis for UBE2C. (**H**) Survival analysis for UBE2T.

**Figure 7:**
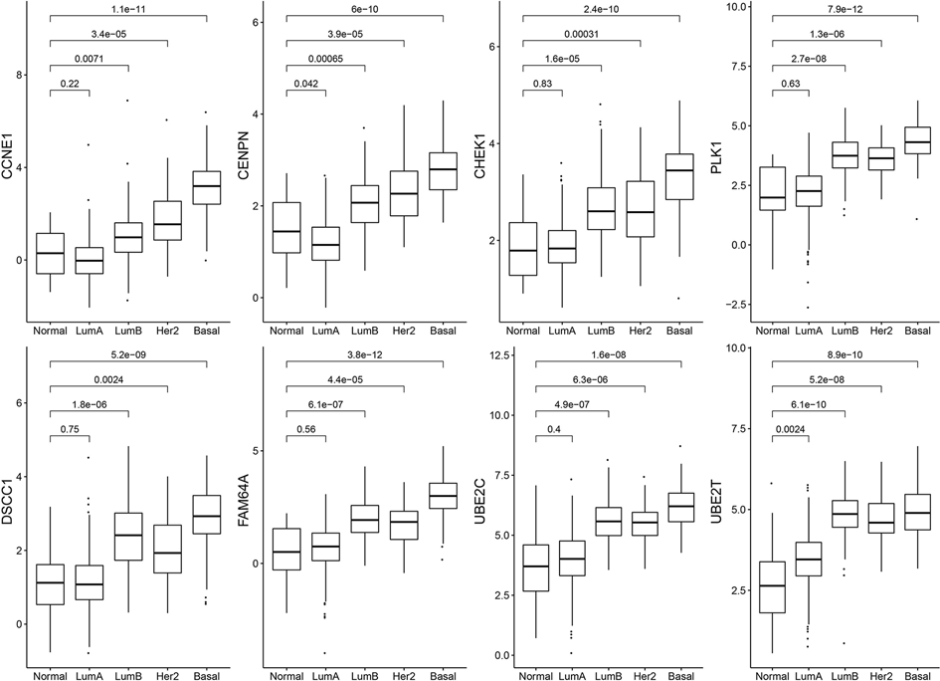
Boxplots showing the expression of hub genes among PAM50 subtypes The *P*-values were calculated using Wilcoxon rank-sum test. (**A**) The expression of CCNE1 among PAM50 subtypes. (**B**) The expression of CENPN among PAM50 subtypes. (**C**) The expression of CHEK1 among PAM50 subtypes. (**D**) The expression of PLK1 among PAM50 subtypes. (**E**) The expression of DSCC1 among PAM50 subtypes. (**F**) The expression of FAM64A among PAM50 subtypes. (**G**) The expression of UBE2C among PAM50 subtypes. (**H**) The expression of UBE2T among PAM50 subtypes.

During the research process, a total of 96 hub genes were identified under GS > 0.2 and MM > 0.8 in the blue module. GEPIA was performed to analyze the overall survival and *P*<0.05 was considered to be statistically significant. Eight genes showed significant associations with the prognosis of patients (Figure 6) and showed increased expression with the aggressive degrees (Figure 7). These fundamental genes include Cyclin E1 (CCNE1), Centromere Protein N (CENPN), Checkpoint Kinase 1 (CHEK1), Polo-Like Kinase 1 (PLK1), DNA Replication and Sister Chromatid Cohesion 1 (DSCC1), PICALM Interacting Mitotic Regulator (Family with Sequence Similarity 64, Member A, FAM64A), Ubiquitin Conjugating Enzyme E2 C (UBE2C), and Ubiquitin Conjugating Enzyme E2 T (UBE2T).

### Validation of hub genes by independent dataset and qRT-PCR

**Figure 8:**
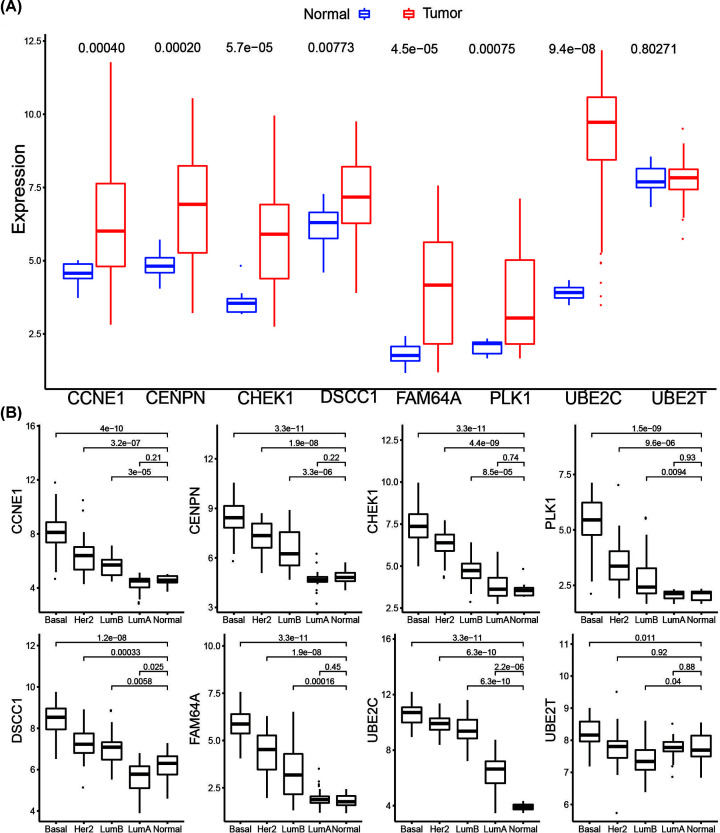
Validation of hub genes in GSE45827 (**A**) Analysis of differential expression in cancer patients compared with normal controls on the basis of GSE45827. (**B**) Boxplots showing the expression of hub genes among PAM50 subtypes in GSE45827. The *P*-values were calculated using Wilcoxon rank-sum test.

**Figure 9:**
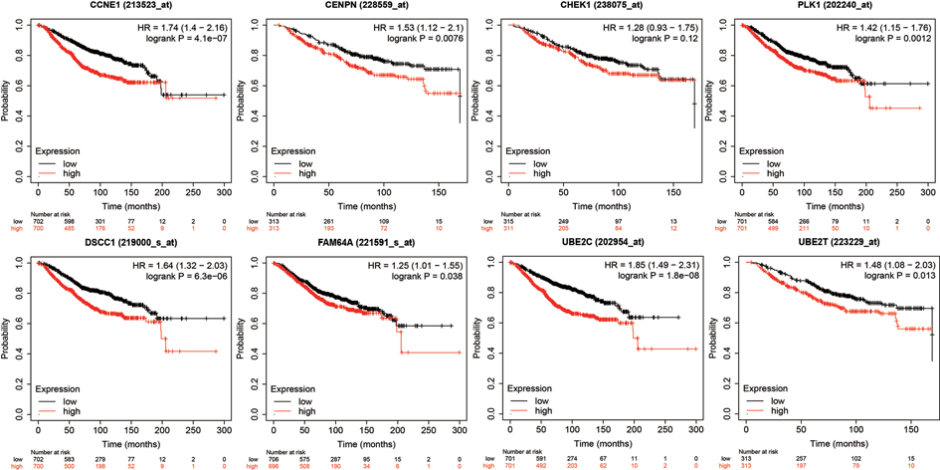
Survival analysis of the hub genes in KM-plotter

**Figure 10:**
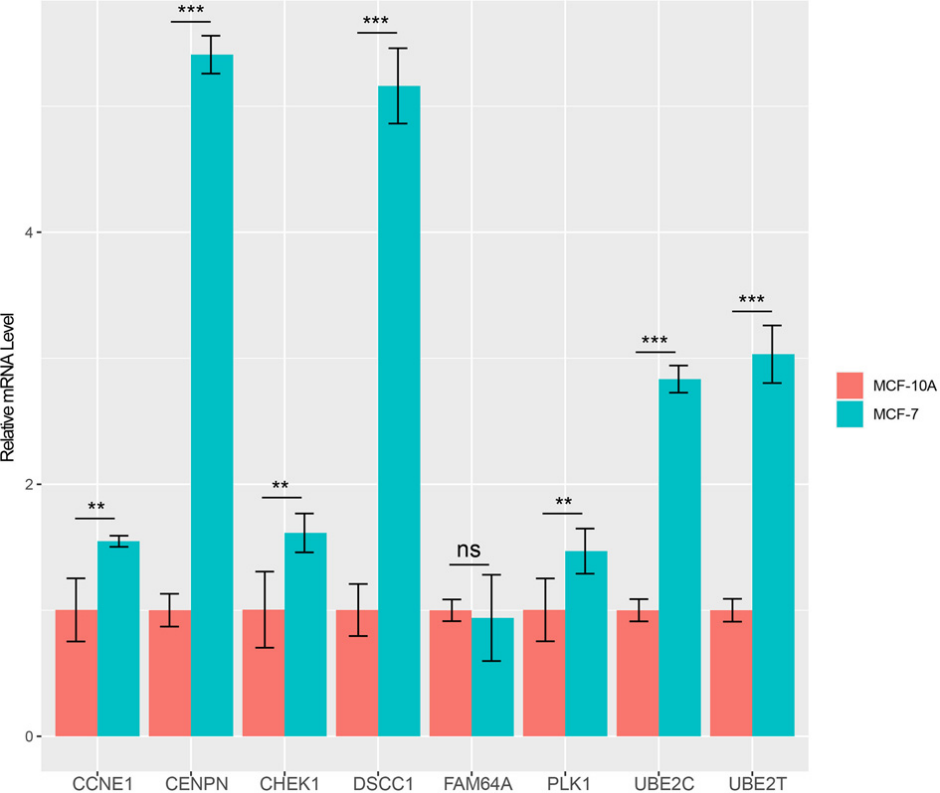
The qPCR results of hub genes in the breast cancer cell line MCF-7 and the normal breast cell line MCF-10A **P<0.01, ***P<0.001, ns not significant.

An independent dataset GSE45827 from GEO database was used to validate the expression status of the eight hub genes. All the eight hub genes showed significant up-regulation in breast cancer compared with normal, except UBE2T (Figure 8A)*.* They also exhibited increased expression with the aggressive degrees in the independent dataset (Figure 8B). The survival analysis of the eight vital genes were validated by Kaplan–Meier Plotter by the microarray datasets which are in accord with the results from the GEPIA using the TCGA data (Figure 9). Besides external datasets validation, we also used qRT-PCR to validate the expression level of hub genes in breast cancer. The results showed the hub genes were significantly up-regulated in breast cancer cell line, except FAM64A (Figure 10).

### Potential functions for each hub gene in breast cancer

**Figure 11:**
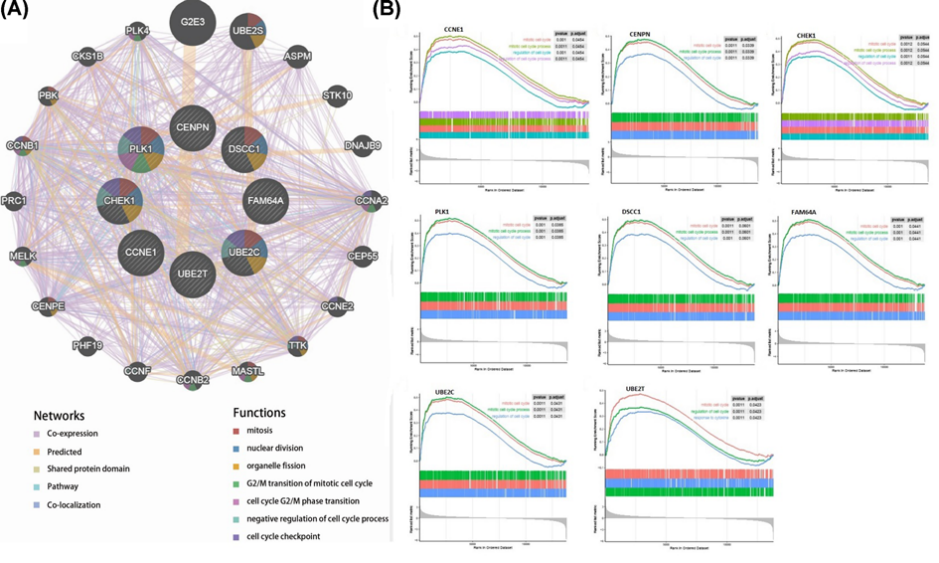
The potential functions for hub genes paticipated (**A**) Protein–protein interaction network (GeneMANIA) of breast cancer-related crucial genes. (**B**) Single-gene GSEA results.

The database of GeneMANIA was used to investigate the functions of the hub genes (Figure 11A). The results showed that these genes were associated with mitosis (FDR = 7.47e-10), nuclear division (FDR = 1.28e-08), organelle fission (FDR = 2.16e-08), G_2_/M transition of mitotic cell cycle (FDR = 4.99e-06), cell cycle G_2_/M phase transition (FDR = 4.99e-06), negative regulation of cell cycle process (FDR = 3.42e-05), and cell cycle checkpoint (FDR = 3.42e-05). For the functions of each hub gene in breast cancer, GO, KEGG pathway enrichment analysis, and GSEA were performed in GSE45827 and the results revealed that the major biological processes and KEGG pathways where hub gene participated were cell cycle, DNA replication, cell cycle checkpoint, and other cell cycle functions closely associated with the proliferation of tumor cells (Table 2 and Figure 11B).

**Table 2:** Functions of single hub gene involved in for breast cancer

ID	Description	CCNE1	CENPN	CHEK1	PLK1	DSCC1	FAM64A	UBE2C	UBE2T
**hsa04110**	**Cell cycle**	1.19E-14	1.92E-11	2.29E-14	8.18E-14	6.20E-14	1.52E-15	4.72E-17	1.80E-11
**hsa03030**	**DNA replication**	1.23E-05	9.53E-04	2.33E-06	1.88E-07	1.56E-04	1.94E-05	2.59E-05	7.12E-05
**hsa04114**	**Oocyte meiosis**	1.22E-05	5.19E-04	8.54E-05	1.28E-05	4.36E-05	3.72E-04	4.59E-06	2.78E-05
**hsa04512**	**ECM–receptor interaction**	3.23E-02	1.16E-03	4.79E-03	2.86E-04	4.62E-03	1.48E-03	1.08E-03	1.44E-03
**hsa04115**	**p53 signaling pathway**	6.42E-04	5.19E-04	1.11E-02	8.75E-04	6.23E-03	1.48E-03	2.68E-05	5.81E-03
**hsa04510**	**Focal adhesion**	NA	3.25E-02	4.94E-02	3.88E-02	NA	1.58E-02	NA	4.62E-02
**GO:0000280**	**Nuclear division**	4.51E-17	6.43E-15	9.13E-16	1.05E-16	2.76E-18	1.05E-14	8.97E-21	3.23E-16
**GO:0140014**	**Mitotic nuclear division**	6.69E-17	2.35E-15	9.08E-16	1.56E-16	6.94E-20	2.90E-14	2.58E-19	6.36E-16
**GO:0006260**	**DNA replication**	1.83E-14	1.01E-08	1.27E-14	4.46E-14	1.92E-10	4.75E-13	4.73E-12	1.30E-10
**GO:0000075**	**Cell cycle checkpoint**	2.32E-08	3.70E-05	1.26E-08	1.52E-07	7.83E-07	5.27E-06	4.16E-09	6.53E-06
**GO:0044843**	**Cell cycle G_1_/S phase transition**	5.90E-08	5.44E-07	2.55E-07	3.41E-07	1.63E-09	1.41E-08	1.87E-09	1.34E-08
**GO:0000082**	**G_1_/S transition of mitotic cell cycle**	5.97E-08	5.44E-07	2.23E-07	2.93E-07	1.98E-09	2.68E-08	6.44E-09	4.07E-08

## Discussion

Breast cancer is a variety of cancers with high biological heterogeneity. The expression level of estrogen receptors (ERs), progesterone receptors (PRs), and human epidermal growth factor receptors 2 (HER2) can affect the survival rate of patients, and may help guide patient treatment and predict prognosis of breast cancer. Although breast cancer treatment measures have ameliorated in the last few decades, the capability to treat the different subtypes, especially Her2 positive and basal-like breast cancer, is still limited by the lack of precise molecular targets. Therefore, it is urgent to continue to explore the molecular mechanisms involved in various subtypes of breast cancer. In the present study, we applied gene transcriptional datasets from the TCGA database to identify potential biomarkers for the cancer subtypes and validated the results using the external datasets and qRT-PCR experiment.

WGCNA was performed to explore gene co-expression modules associated with breast cancer subtypes. With a total of 6315 DEGs being evaluated to construct a co-expression network, 7 co-expressed modules were identified. The blue and turquoise modules in the present study were found to have the highest association with tumor subtypes. Further gene functional enrichment of each module revealed that the blue module is involved in functions of cell cycle, DNA replication, p53 signaling pathway and other functions commonly associated with cancer. Additionally, 96 hub genes with high connectivity were screened out from the blue module. Among them, eight genes including *CCNE1, CENPN, CHEK1, PLK1, DSCC1, FAM64A, UBE2C*, and *UBE2T*, were significantly negatively associated with the overall patient survival with log-rank test *P*-value <0.05 and exhibited increased expression with the aggressive degrees. The independent data and qRT-PCR showed that they were significantly up-expressed and associated with poor outcome in breast cancer except UBE2T and FAM64A. Furthermore, single gene functional analysis for each hub gene in breast cancer was conducted and revealed that they were all involved in cycle cycle process whose aberration was reported as the hallmarks of cancer [19] and were validated to be anti-cancer therapeutic targets [20]. CCNE1 is an oncogene and is one of the members of the cyclin family. Genes in this family are marked by the dramatic periodicity in protein abundance through the cell cycle. CCNE1 combines and activates the expression of CDK2 to regulate the cell cycle G_1_/S transition. Overexpression of this gene has been observed in many tumors, which results in chromosome instability, and thus may contribute to tumorigenesis [21]. Nakayama et al. [22] reported that the amplification of gene CCNE1 is related to poor survival rates in ovarian cancer patients. In the present study, CCNE1 is significantly overexpressed in luminal B, Her2 positive, and basal-like subtypes compared with normal samples (Figure 8), which suggests that the expression of CCNE1 is associated with the relatively aggressive degrees of cancer. Han et al. [23] showed that miR-497 and miR-34a can down-regulate CCNE1 by targeting the 3′-UTR of CCNE1 in lung cancer. CENPN forms part of the nucleosome-associated complex and is important for kinetochore assembly. This protein is bound to kinetochores during S phase and G_2_, and recruits other proteins to the centromere. CENPN is an important predictor for both breast carcinoma recurrence and mortality among smokers. Elevated expression of this gene is associated with significantly increased mortality and risk of recurrence in smokers in contrast with non-smokers [24]. CHEK1 is a cancer-related gene. Protein encoded by CHEK1 is a member of the Ser/Thr protein kinase family. It is necessary for checkpoint-mediated cell cycle arrest in response to DNA damage or the presence of unreplicated DNA. This protein can integrate signals from two cell cycle proteins (ATM and ATR) related to DNA damage responses, that also associate with chromatin in meiotic prophase I [25]. Tarek et al. [26] reported that at the mRNA level, high CHEK1 was associated with poor survival in the whole cohort of breast cancer and in the ER+ subgroup according to data analysis. PLK1 is a main mitotic kinase playing a pivotal role in the eukaryotic cells division [27]. Many researchers have demonstrated that PLK1 is highly expressed in various tissues for tumor, such as breast cancer, ovarian cancer, and prostate cancer [28,29]. A high expression of PLK1 is associated with aggressive characteristics, such as invasion of vascular, proliferative activity markers and detectable ERs shortage [29,30]. Similarly, analysis revealed that PLK1 was significantly up-regulated in the subtypes except luminal A, which is less aggressive. King et al. [31] showed a significant association between increased PLK1 levels in breast cancer cells and clinical outcome. PLK1 expression was associated almost exclusively with tumor grade. The survival data clearly validated that patients expressing increased PLK1 levels appear significantly reduced survival, which is in accordance with the finding that high levels of PLK1 are associated with poor patient outcome. DSCC1 is a component of an alternative replication factor C complex that loads proliferating cell nuclear antigens on to DNA during S-phase of the cell cycle [32]. In the present study, DSCC1 is significantly up-expressed in each breast cancer subtype except luminal A, compared with normal samples. This fact indicates that overexpression probably promotes the cell cycle transition and cell division, and consequently promotes cell growth. UBE2C is a cellular proto-oncogene and is essential for the mitotic cyclins destruction, for the anaphase-promoting complex regulation, and for progression of cell cycle [33]. Overexpressed UBE2C can cause mis-segregation of chromosome and the cell cycle profile alteration facilitating the proliferation of cell [34]. UBE2C overexpressed in various human cancers, including breast cancer and is correlated with tumor malignancy [35–37]. Although, FAM64A and UBE2T were not validated both in independent dataset and qRT-PCR, they still play important roles in cancer. FAM64A is reported to be associated with several cancers [38–40], aberrant expression was found in tumor samples, and was significantly correlated with patients’ survival in multiple cancers, including breast cancer [41]. FAM64A can be combined with Twist1 to form a complex, which could bind with an E-cadherin promoter to inhibit the expression of E-cadherin, and thus promote the process of cell epithelial–mesenchymal transition. It was specifically up-regulated in triple-negative breast cancer (TNBC) which showed more aggressive clinical behavior [42]. To our best knowledge that MCF-7 cell line had a low degree of malignancy and the expression level was not significantly changed by qRT-PCR, we speculated that FAM64A was associated with the aggressive degree in breast cancer. UBE2T engage in the DNA repair pathway through catalyzing the Fanconi Anemia Complex monoubiquitination [43]. UBE2T has been reported to be associated with cancer progression and poor outcomes in several solid tumors [44,45]. It has been demonstrated that UBE2T has a crucial role in the regulation of BRCA1 in breast cancer [46]. Overexpressed UBE2T can facilitate cell cycle progression and avoid DNA repair by inducing the degradation of key regulators of functions such as p21 and BRCA1 [47]. Although eight hub genes were identified to be potential prognosis biomarkers for breast cancers, the further experiments need to be conducted to investigate the mechanisms of them in the cell cycle process in breast cancer.

## Conclusion

Using the WGCNA method, an important gene co-expression module blue was identified which is closely associated with different subtypes of breast cancer. In addition, eight hub genes of CCNE1, CENPN, CHEK1, PLK1, DSCC1, FAM64A, UBE2C, and UBE2T were identified and validated to be the potential prognostic and therapeutic biomarkers for breast cancer subtypes.

## Supplementary Material

Supplementary Figures S1-S2 and Table S1

## Data Availability

The RNA-seq datasets were downloaded from TCGA; http://cancergenome.nih.gov. The GSE45827 was downloaded from GEO website; https://www.ncbi.nlm.nih.gov/gds/?term=GSE45827. Other data are supplied in the paper and the supplementary data.

## Abbreviations

CCNE1: cyclin E1
cDNA: complementary DNA
CENPN: centromere protein N
CHEK1: checkpoint kinase 1
DEG: differentially expressed gene
DMEM: Dulbecco’s modified eagle’s medium
DSCC1: DNA replication and sister chromatid cohesion 1
ER: estrogen receptor
FAM64A: family with sequence similarity 64, member A
FDR: false discovery rate
GEPIA: gene expression profiling interactive analysis
GO: gene ontology
GS: gene significance
Her2: human epidermal growth factor receptor 2
ME: module eigengene
MM: module membership
PLK1: polo-like kinase 1
qRT-PCR: quantitative real-time PCR
TCGA: The Cancer Genome Atlas
UBE2C: ubiquitin conjugating enzyme E2 C
UBE2T: ubiquitin conjugating enzyme E2 T
WGCNA: weighted gene co-expression network analysis

## References

[1] Andrew M.G. et al. (2015) Effects of age on the detection and management of breast cancer. Cancers 7, 908–929 26010605 10.3390/cancers7020815 PMC4491690

[2] Liu M.C. et al. (2015) PAM50 gene signatures and breast cancer prognosis with adjuvant anthracycline- and taxane-based chemotherapy: correlative analysis of C9741 (Alliance). NPJ Breast Cancer 2, 15023 10.1038/npjbcancer.2015.23 28691057 PMC5501351

[3] Parker J.S. et al. (2009) Supervised risk predictor of breast cancer based on intrinsic subtypes. J. Clin. Oncol. 27, 1160–1167 10.1200/JCO.2008.18.1370 19204204 PMC2667820

[4] Ruan J., Dean A.K. and Zhang W. (2010) A general co-expression network-based approach to gene expression analysis: comparison and applications. BMC Syst. Biol. 4, 8 10.1186/1752-0509-4-8 20122284 PMC2829495

[5] Liu H. et al. (2020) Oncogenic network and hub genes for natural killer/T-cell lymphoma utilizing WGCNA. Front. Oncol. 10, 223 10.3389/fonc.2020.00223 32195177 PMC7066115

[6] Du B.S. et al. (2020) WGCNA screening of prognostic markers in medulloblastoma. Zhonghua Yi Xue Za Zhi 100, 460–464 32146771 10.3760/cma.j.issn.0376-2491.2020.06.013

[7] Wang H. et al. (2020) Identification of gene modules and hub genes in colon adenocarcinoma associated with pathological stage based on WGCNA analysis. Cancer Genet. 242, 1–7 10.1016/j.cancergen.2020.01.052 32036224

[8] Guo Y. et al. (2019) Identification of key pathways and genes in different types of chronic kidney disease based on WGCNA. Mol. Med. Rep. 20, 2245–2257 31257514 10.3892/mmr.2019.10443 PMC6691232

[9] Cancer Genome Atlas Research Network (2013) The Cancer Genome Atlas Pan-Cancer analysis project. Nat. Genet. 45, 1113–1120 10.1038/ng.2764 24071849 PMC3919969

[10] Robinson M.D., McCarthy D.J. and Smyth G.K. (2010) edgeR: a Bioconductor package for differential expression analysis of digital gene expression data. Bioinformatics 26, 139–140 10.1093/bioinformatics/btp616 19910308 PMC2796818

[11] Li J. et al. (2012) Normalization, testing, and false discovery rate estimation for RNA-sequencing data. Biostatistics 13, 523–538 10.1093/biostatistics/kxr031 22003245 PMC3372940

[12] Yip A.M. and Horvath S. (2007) Gene network interconnectedness and the generalized topological overlap measure. BMC Bioinformatics 8, 22 10.1186/1471-2105-8-22 17250769 PMC1797055

[13] Ravasz E. et al. (2002) Hierarchical organization of modularity in metabolic networks. Science 297, 1551–1555 10.1126/science.1073374 12202830

[14] Yu G. et al. (2012) clusterProfiler: an R package for comparing biological themes among gene clusters. OMICS 16, 284–287 10.1089/omi.2011.0118 22455463 PMC3339379

[15] Tang Z. et al. (2017) GEPIA: a web server for cancer and normal gene expression profiling and interactive analyses. Nucleic Acids Res. 45, W98–W102 10.1093/nar/gkx247 28407145 PMC5570223

[16] Gyorffy B. et al. (2010) An online survival analysis tool to rapidly assess the effect of 22,277 genes on breast cancer prognosis using microarray data of 1,809 patients. Breast Cancer Res. Treat. 123, 725–731 10.1007/s10549-009-0674-9 20020197

[17] Warde-Farley D. et al. (2010) The GeneMANIA prediction server: biological network integration for gene prioritization and predicting gene function. Nucleic Acids Res. 38, W214–W220 10.1093/nar/gkq537 20576703 PMC2896186

[18] Zuberi K. et al. (2013) GeneMANIA prediction server 2013 update. Nucleic Acids Res. 41, W115–W122 10.1093/nar/gkt533 23794635 PMC3692113

[19] Dominguez-Brauer C. et al. (2015) Targeting mitosis in cancer: emerging strategies. Mol. Cell 60, 524–536 10.1016/j.molcel.2015.11.006 26590712

[20] Chi J.J. et al. (2019) A novel strategy to block mitotic progression for targeted therapy. EBiomedicine 49, 40–54 10.1016/j.ebiom.2019.10.013 31669221 PMC6945239

[21] Zhao Z.M. et al. (2019) CCNE1 amplification is associated with poor prognosis in patients with triple negative breast cancer. BMC Cancer 19, 96 10.1186/s12885-019-5290-4 30665374 PMC6341717

[22] Nakayama N. et al. (2010) Gene amplification CCNE1 is related to poor survival and potential therapeutic target in ovarian cancer. Cancer 116, 2621–2634 20336784 10.1002/cncr.24987

[23] Han Z. et al. (2015) miR-497 and miR-34a retard lung cancer growth by co-inhibiting cyclin E1 (CCNE1). Oncotarget 6, 13149–13163 10.18632/oncotarget.3693 25909221 PMC4537005

[24] Andres S.A. et al. (2015) Interaction between smoking history and gene expression levels impacts survival of breast cancer patients. Breast Cancer Res. Treat. 152, 545–556 10.1007/s10549-015-3507-z 26202054

[25] Zhang Y. and Hunter T. (2014) Roles of Chk1 in cell biology and cancer therapy. Int. J. Cancer 134, 1013–1023 10.1002/ijc.28226 23613359 PMC3852170

[26] Abdel-Fatah T.M. et al. (2015) Untangling the ATR-CHEK1 network for prognostication, prediction and therapeutic target validation in breast cancer. Mol. Oncol. 9, 569–585 10.1016/j.molonc.2014.10.013 25468710 PMC5528699

[27] Barr F.A., Sillje H.H. and Nigg E.A. (2004) Polo-like kinases and the orchestration of cell division. Nat. Rev. Mol. Cell Biol. 5, 429–440 10.1038/nrm1401 15173822

[28] Takai N. et al. (2005) Polo-like kinases (Plks) and cancer. Oncogene 24, 287–291 10.1038/sj.onc.1208272 15640844

[29] Weichert W. et al. (2005) Polo-like kinase isoforms in breast cancer: expression patterns and prognostic implications. Virchows Arch. 446, 442–450 10.1007/s00428-005-1212-8 15785925

[30] Wolf G. et al. (2000) Polo-like kinase: a novel marker of proliferation: correlation with estrogen-receptor expression in human breast cancer. Pathol. Res. Pract. 196, 753–759 10.1016/S0344-0338(00)80107-7 11186170

[31] King S.I. et al. (2012) Immunohistochemical detection of Polo-like kinase-1 (PLK1) in primary breast cancer is associated with TP53 mutation and poor clinical outcom. Breast Cancer Res. 14, R40 10.1186/bcr3136 22405092 PMC3446374

[32] Xie X.W. et al. (2018) Effect of upregulated DNA replication and sister chromatid cohesion 1 expression on proliferation and prognosis in hepatocellular carcinoma. Chin. Med. J. (Engl.) 131, 2827–2835 30511685 10.4103/0366-6999.246076 PMC6278189

[33] Okamoto Y. et al. (2003) UbcH10 is the cancer-related E2 ubiquitin-conjugating enzyme. Cancer Res. 63, 4167–4173 12874022

[34] van Ree J.H. et al. (2010) Overexpression of the E2 ubiquitin-conjugating enzyme UbcH10 causes chromosome missegregation and tumor formation. J. Cell Biol. 188, 83–100 10.1083/jcb.200906147 20065091 PMC2812857

[35] Chou C.P. et al. (2014) Ubiquitin-conjugating enzyme UBE2C is highly expressed in breast microcalcification lesions. PLoS ONE 9, e93934 10.1371/journal.pone.0093934 24699941 PMC3974821

[36] Pallante P. et al. (2005) UbcH10 overexpression may represent a marker of anaplastic thyroid carcinomas. Br. J. Cancer 93, 464–471 10.1038/sj.bjc.6602721 16106252 PMC2361574

[37] Wagner K.W. et al. (2004) Overexpression, genomic amplification and therapeutic potential of inhibiting the UbcH10 ubiquitin conjugase in human carcinomas of diverse anatomic origin. Oncogene 23, 6621–6629 10.1038/sj.onc.1207861 15208666

[38] Hashimoto K. et al. (2017) Fam64a is a novel cell cycle promoter of hypoxic fetal cardiomyocytes in mice. Sci. Rep. 7, 4486 10.1038/s41598-017-04823-1 28667270 PMC5493652

[39] Yamada Y. et al. (2018) Regulation of antitumor miR-144-5p targets oncogenes: direct regulation of syndecan-3 and its clinical significance. Cancer Sci. 109, 2919–2936 10.1111/cas.13722 29968393 PMC6125479

[40] Jiao Y. et al. (2019) Aberrant FAM64A mRNA expression is an independent predictor of poor survival in pancreatic cancer. PLoS ONE 14, e0211291 10.1371/journal.pone.0211291 30695070 PMC6351057

[41] Zhang J. et al. (2019) Up-regulation of FAM64A promotes epithelial-to-mesenchymal transition and enhances stemness features in breast cancer cells. Biochem. Biophys. Res. Commun. 513, 472–478 10.1016/j.bbrc.2019.03.207 30979502

[42] Zhang C. et al. (2014) Integrated analysis of expression profiling data identifies three genes in correlation with poor prognosis of triple-negative breast cancer. Int. J. Oncol. 44, 2025–2033 10.3892/ijo.2014.2352 24676531

[43] Machida Y.J. et al. (2006) UBE2T is the E2 in the Fanconi anemia pathway and undergoes negative autoregulation. Mol. Cell 23, 589–596 10.1016/j.molcel.2006.06.024 16916645

[44] Luo C. et al. (2017) UBE2T knockdown inhibits gastric cancer progression. Oncotarget 8, 32639–32654 10.18632/oncotarget.15947 28427240 PMC5464816

[45] Wen M. et al. (2015) Elevated expression of UBE2T exhibits oncogenic properties in human prostate cancer. Oncotarget 6, 25226–25239 10.18632/oncotarget.4712 26308072 PMC4694827

[46] Ueki T. et al. (2009) Ubiquitination and downregulation of BRCA1 by ubiquitin-conjugating enzyme E2T overexpression in human breast cancer cells. Cancer Res. 69, 8752–8760 10.1158/0008-5472.CAN-09-1809 19887602

[47] Perez-Pena J. et al. (2017) Ubiquitin-conjugating enzyme E2T (UBE2T) and denticleless protein homolog (DTL) are linked to poor outcome in breast and lung cancers. Sci. Rep. 7, 17530 10.1038/s41598-017-17836-7 29235520 PMC5727519

