# Supplementary material for: Identifying breast cancer subtypes associated modules and biomarkers by integrated bioinformatics analysis
Source: Biosci Rep. 2021 Jan 8;41(1):BSR20203200. doi: 10.1042/BSR20203200 (PMC7796196; doi:10.1042/BSR20203200)
Supplement: Supplementary Figures S1-S2 and Table S1 [file BSR-2020-3200_supp.pdf]

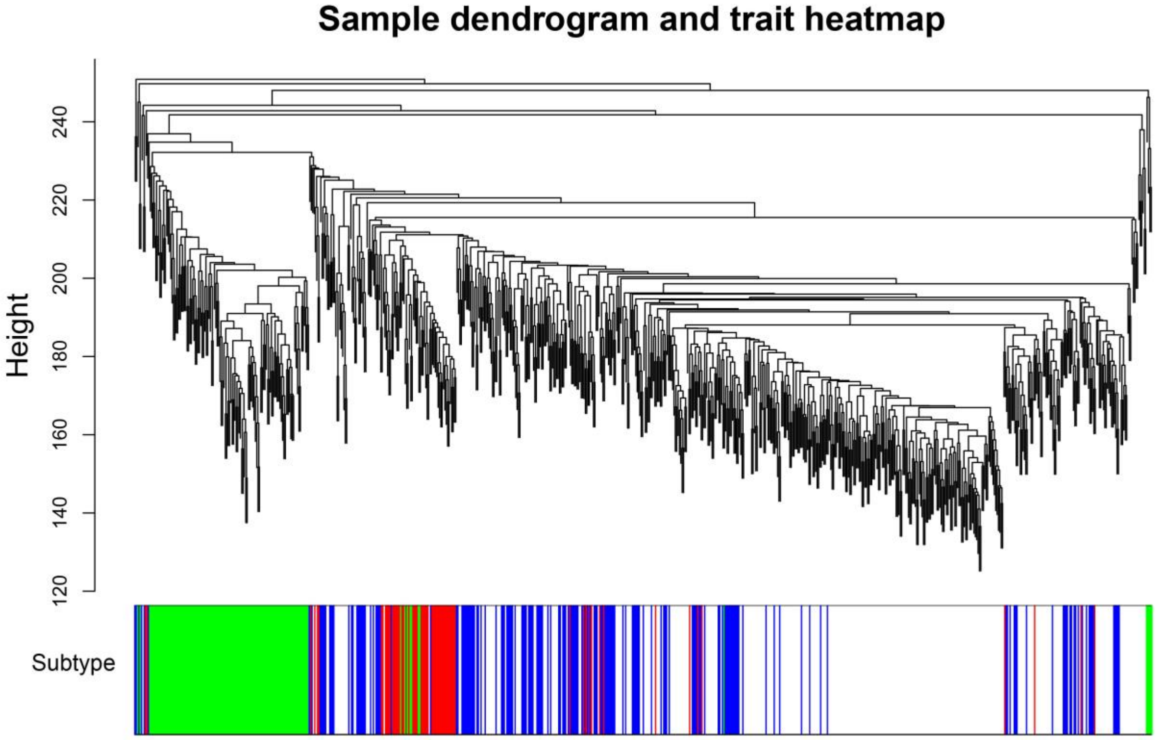


**Supple Figure 1.** The clustering was based on the expression data of DEGs, which contained 373 Luminal A (white), 177 Luminal B (blue), 65 Her2 positive (red) and 130 Basal like (green) subtype samples.

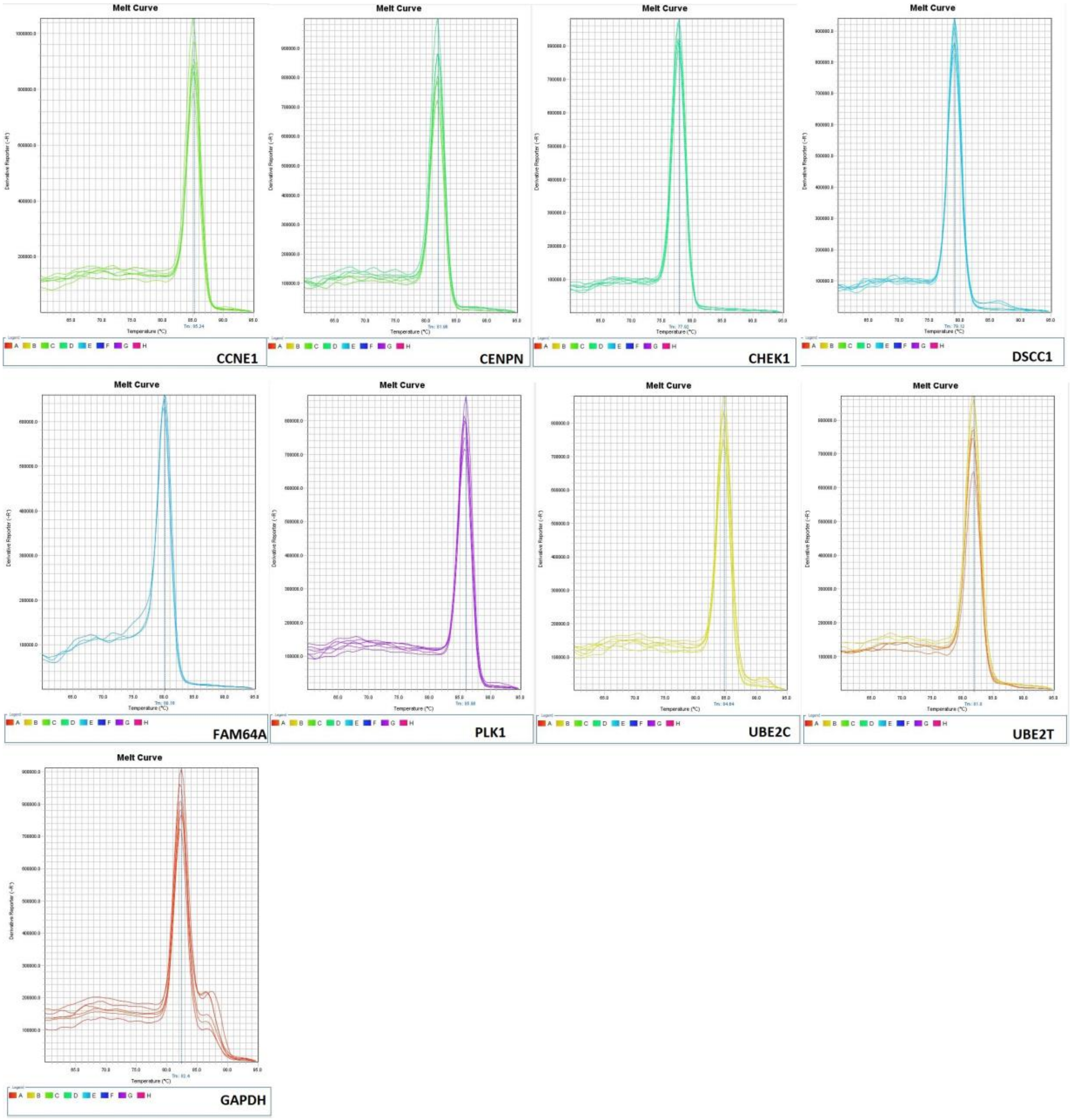


**Supple Figure 2.** The melting curves for hub genes in qRT-PCR.

**Table 1. The primer sequences (5´ to 3´) used for gene amplification.**

| Gene | Upstream primer(5'-3') | Downstream primer(5'-3') |
|---|---|---|
| CCNE1 | GGAAGAGGAAGGCAAACGTG | GCAATAATCCGAGGCTTGCA |
| CENPN | TGAGGAGTGAGACTGCAGGA | CCCAGGCCTTCAGGATTGTT |
| CHEK1 | ATATGAAGCGTGCCCTAGACT | TGCCTATGTCTGGCTCTATTCTG |
| PLK1 | GCTTTGCCAAGTGCTTCGAG | AATCCTACGACGTGCTGGTG |
| DSCC1 | TCCATATGAAGGACCTGACAGT | CCGAGTTCCTGAAGGCATGT |
| FAM64A | GCAGACTTGAACCGTTGCTG | TGTTGGTGAGGCATGCTGAT |
| UBE2C | AGCAGCTGGAACAAACCCAA | AAGACGACACAAGGACAGGC |
| UBE2T | ATGTTAGCCACAGAGCCACC | GGTGTGTTGGCTCCACCTAA |
| GAPDH | AGGGCTGCTTTTAACTCTGGT | CCCCACTTGATTTTGGAGGGA |
